# Ag-Decorated Iron Oxides-Silica Magnetic Nanocomposites with Antimicrobial and Photocatalytic Activity

**DOI:** 10.3390/nano12244452

**Published:** 2022-12-15

**Authors:** Viorica Muşat, Lenuța Crintea (Căpăţână), Elena-Maria Anghel, Nicolae Stănică, Irina Atkinson, Daniela Cristina Culiţă, Liliana Baroiu, Nicolae Țigău, Alina Cantaragiu Ceoromila, Andreea-Veronica Botezatu (Dediu), Oana Carp

**Affiliations:** 1 Laboratory of Chemical Nanotechnologies-Center of Nanostructures and Functional Materials LNC-CNMF, Dunărea de Jos” University of Galati, 47 Domnească Street, 800008 Galati, Romania; 2 Institute of Physical Chemistry “Ilie Murgulescu” of Romanian Academy, Spl. Independenţei 202, 060021 Bucharest, Romania; 3 Faculty of Medicine and Pharmacy, “Dunărea de Jos” University of Galati, 47 Domnească Street, 800008 Galati, Romania; 4 Department of Physical-Chemistry and Environment, “Dunărea de Jos” University of Galati, 47 Domnească Street, 800008 Galati, Romania; 5 Department of Applied Sciences, Cross-Border Faculty, “Dunărea de Jos” University of Galati, 47 Domnească Street, 800008 Galati, Romania

**Keywords:** iron oxides-silica composite, Ag-decorated, co-precipitation, ultrasound-assisted sol-gel, superparamagnetic nanoparticles, antimicrobial, photocatalysis

## Abstract

Nanotechnology offers unlimited possibilities for creating effective hybrid materials, which combine functional performance in environment depollution and antimicrobial defense with a lack of toxicity, biocompatibility, biodegradability, and natural availability. This paper presents the silver effect on photocatalytic and antibacterial activities of double-coated iron oxide nanoparticles (NPs), Fe_3_O_4_@SiO_2_/ZnO-Ag. The structural, morphological, and textural information of the, core–shell iron oxides-based superparamagnetic nanoparticles (IOMNPs) decorated with 5% Ag by ultrasound-assisted synthesis were evaluated by scanning electron microscopy with energy dispersive spectroscopy (SEM-EDX), X-ray diffraction, Raman spectroscopy, and Brunauer–Emmett–Teller physisorption measurements. Although two synthesis temperatures of 95 and 80 °C were used for the co-precipitated iron oxide cores, the XRD patterns revealed the formation of a single magnetite, Fe_3_O_4_, phase. The sorption–photocatalytic activities under dark and UV irradiation encountered a maximum removal efficiency of the MB (90.47%) for the Fe_3_O_4_@SiO_2_/ZnO-Ag sample with iron oxide core obtained at 80 °C. The rate constant for the second-order kinetics was 0.0711 min^−1^ for 2 h, and the correlation coefficient R^2^ closed to unity. Two samples with Ag-decorated hybrid SiO_2_/ZnO shell and hierarchically interconnected porous structure with large surface area (328.8 and 342.5 m^2^g^−1^) exhibited the best disk diffusion antimicrobial activity against four microorganisms, especially gram-positive *Staphylococcus aureus*.

## 1. Introduction

The accelerated development of the industrial, agricultural, transportation, and other sectors has created a major risk of planet pollution, as well as an urgent need to remedy and mitigate its effects. Furthermore, in the last years, microbial and viral contamination has become a widespread phenomenon, with major consequences on human health, society, and economy. Currently, it is looking for effective materials and methods for depollution [1,2,3,4,5,6] and antimicrobial defense [7,8,9,10,11], obtained through environment friendly practices. Nanotechnology offers multiple possibilities. In this context, the scientific literature presents the synthesis of increasingly complex hybrid materials that combine functional performance with a lack of toxicity, biocompatibility, and biodegradability, as well as natural availability.

Recently, metal oxide-based materials with magnetic properties that allow for their magnetic manipulation under external magnetic fields have been applied in the research fields involving their photocatalytic and antimicrobial properties [7]. Thus, iron oxide-based magnetic nanoparticles (IOMNPs), especially magnetite (Fe_3_O_4_) and maghemite (γ-Fe_2_O_3_), are among the most used metal oxides, with low toxicity, good biocompatibility, biodegradability, availability, and recyclability [1,7,8]. To prevent nanoparticle agglomeration, oxidation, and/or degradation under harsh environments and repeated use, the ferromagnetic catalysts of magnetite are often protected with a SiO_2_ layer in so-called core–shell structures [4,8,9,10]. Additionally, the SiO_2_ shell allows further functionalization of the iron oxide nanoparticles (IONPs) by its silanol groups (Si-OH) [10,12].

Further capping/covering of IONPs with TiO_2_ or ZnO NPs is required to improve the core–shell performance for a wide range of applications [8,9,12,13,14,15]. Thus, semiconductive ZnO nanoparticles (NPs) have the advantage of size- and morphology-dependent wide bandgap energy (3.37 eV in bulk), high excitation energy (60 meV), and antibacterial activity [14,16,17]. The 0D, 1D, 2D, or 3D ZnO nanostructures [13,14,18] are well-known for their ability to absorb UV radiation and manifest optoelectronic effects, with applications in photovoltaic cells [16], radiation and gas sensors [13,19], biomedical devices and pharmaceuticals [18,19,20,21], food industry [22], but also membrane separation [23,24], etc. Double-covered IONPs (Fe_3_O_4_@SiO_2_@ZnO) were also obtained by physical and chemical methods [4,23]. Ultrasound-assisted co-precipitation and sol-gel methods are the most known methods for the synthesis of core–shell IONPs with predetermined morphology, crystallinity, and size [1,4].

The main drawback of using semiconductor metal oxides, such as TiO_2_, ZnO, and Fe_3_O_4_, in photocatalysis applications is the fast rate of exciton (photogenerated electron-hole pair) recombination, which hinders their industrial-scale applications [25]. By doping iron oxide with transitional or noble metals [1,2,8,25,26,27], graphene oxide [28], and nonmetals (N or S [29,30]), the exciton recombination is tuned, leading to a substantial improvement of the photocatalytic stability and efficiency. Ferrite phases, Fe_3-x_M_x_O_4_, were obtained by doping magnetite with transition metals (M = Cu, Mn, Cr, Ni, Co, Zn, etc.) [31]. Additionally, metal dopants can adjust the band gap width and visible (VIS) adsorption, hence making it possible to obtain efficient nano-photocatalysts from UV to VIS light [32] and antimicrobial and antiviral agents, based on IOMNPs. Some challenges were encountered for the metal-doped magnetite photocatalysts, very likely due to their surface structure (metal self-aggregation or dopant segregation, as well as metal nanoparticle polydispersity [31]), which requires further clarification.

The selection of a metal dopant and synthesis route for the metal decoration of IONPs considerably influences the properties and applications of the obtained materials. While Cu and Ni enhanced magnetite activity, Cr improved their thermal stability [32]. Unlike the noble metal majority, the inexpensive silver is one of the most stable noble metals with good surface plasmon resonance, which is often used for metal-decorated semiconductor metal oxides (SiO_2_, ZnO, TiO_2_, etc.) [23,24,25,26,27,28,29,30,31,32,33,34,35]. The latter nanocomposites have been used as substrates for surface-enhanced Raman spectroscopy (SERS), which enables the very accurate detection of material traces down to a single molecule [35]. So far, high costs and poor stability of the SERS substrates have prevented the use of SERS as a routine analytical tool. Han et al. [36] reported obtaining Fe_3_O_4_/SiO_2_/ZnO/Ag as SERS for detecting pollutants. Weak SERS activity of ZnO was improved by Ag NPs through an electromagnetic mechanism, due to surface plasmon resonance [37]. The recyclable SERS substrate of Fe_3_O_4_/SiO_2_/Ag nanoparticles had a thick SiO_2_ layer to shield the magnetite signal in the Raman detection of the dye rhodamine B traces [38]. Moreover, the heterojunction of the ZnO nanowires (NWs) with Ag enabled the separation of the electrons from photogenerated excitons, extending the excitons’ lifetime with improved photocatalytic capability [38].

Although several studies on core–shell structure have been reported in the literature, there are fewer works that refer to ternary systems decorated with silver [8,23,39]. Using bottom-up or self-assembly nanotechnology methods from solution can easily control the doping concentration. Citrate reduction is one of the most known wet chemical methods to obtain noble metal NPs [40,41]. Using the tri-sodium citrate dehydrate as a reducing agent, the capping and pH modifier is less employed for reducing silver nitrate [40,42].

This work focused on coupling the co-precipitation of iron oxide core (Fe_x_O_y_) with an ultrasonic-assisted sol-gel method using commercial ZnO NPs for obtaining the ternary core-hybrid shell structures of Fe_x_O_y_@SiO_2_/ZnO, which were subsequently decorated with Ag NPs. Silver decoration of the Fe_x_O_y_@SiO_2_/ZnO NPs by using tri-sodium citrate dehydrate in reducing silver nitrate is a novel approach. Photocatalytic and antimicrobial activities of the resulting nanomaterials were also assessed in comparison with the ternary silver-free system.

## 2. Materials and Methods

### 2.1. Reagents

Ferric chloride hexahydrate (FeCl_3_·6H_2_O), ferrous sulfate heptahydrate (FeSO_4_∙7H_2_O), tetraethyl orthosilicate (TEOS), silver nitrate (AgNO_3_), trisodium citrate dihydrate (HOC(COONa)(CH_2_COONa)_2_∙2H_2_O), and sodium hydroxide (NaOH) from Sigma-Aldrich (Saint Louis, MI, USA). with analytical quality and without further purification were used as reagents. The ZnO nanoparticles (50 nm) were supplied by Merck (Darmstadt, Germany).

### 2.2. Synthesis of Ag-Decorated IONPs

A detailed description of the two-step synthesis from the solution of the hybrid shell (SiO_2_/ZnO) on the IONPs core, Fe_x_O_y_, was presented in [4]. Co-precipitation of Fe_x_O_y_ core in the air from an aqueous solution of Fe(III): Fe(II), (2:1 at.), at two different temperatures 95 and 80 °C (denominated M1 and M2, respectively) was carried out according to Liu’s method, except for the additives to control particle size distribution [43]. The SiO_2_ shell (S1 shell) was obtained by sol-gel method from TEOS, according to Zhao et al.’s protocol [44]. The hybrid shell of SiO_2_/ZnO (S2) was sol-gel synthesized from TEOS and commercial ZnO NPs (3 wt.%).

In the third step, 5 wt.% silver was decorated on the Fe_x_O_y_@SiO_2_/ZnO NPs by ultrasound-assisted synthesis using silver nitrate and trisodium citrate dihydrate as a reducing agent (6:1 molar ratio) [45]. Briefly, over 0.1 g of MNPs were dispersed in 10 mL of distilled water and heated to 90 °C under ultrasound (US) treatment. Then, 50 mL of AgNO_3_ solution (1 × 10^−3^ M) was added and US stirred for another 5 min. To this solution, 25 mL of sodium citrate (3.5 × 10^−4^ M) was added and stirred for 30 min, during which the solution changed color from white to purple, indicating the formation of Ag NPs. After this, the solution with Ag-decorated MNPs was aged under mechanical stirring at 80 °C for 30 min. Then, the resulting sample was cooled down to room temperature and subjected to centrifugation (9000 rpm). The nanoparticles separated by centrifugation were washed five times with distilled water. The Ag-decorated Fe_x_O_y_@SiO_2_/ZnO NPs with the magnetic cores obtained at 95 °C (M1) and 80 °C (M2) and two types of shells (SiO_2_, S1, and SiO_2_/ZnO, S2, respectively) were named AM(1/2)-S(1/2).

### 2.3. Structural and Morphologic Characterization

SEM-EDX data were collected by means of a JEOL JSM-7500F/FA microscope (Peabody, JEOL Massachusetts, Ltd. USA) on the Ag-decorated IONPs before dispersion onto a glass substrate and sputtered with a 5 nm Au layer.

The X-ray diffraction (XRD) patterns were recorded using Rigaku’s Ultima IV diffractometer (Tokyo, Japan) in parallel beam geometry, with a graphite monochromator operating at 40 kV and 30 mA. The signals were collected with a step size of 0.02° step size, and a scan speed of 2°  min^−1^. XRD data were analyzed using Rigaku’s PDXL software, connected to the ICDD PDF-2 database. The reference intensity ratio (RIR) method was used for quantitative XRD analysis.

The average crystallite size of Fe_3_O_4_ was calculated according to Scherrer’s equation using the most intense peak (311) and the Williamson-Hall method [46]:

(1) $$D=\frac{k\lambda }{\beta \cos\theta }$$

where *k* is Scherrer’s constant (*k* = 0.90), *λ* is the X-ray wavelength (1.5406 Å), *β* is the full width at half maximum of the peak (FWHM), and *θ* is the diffraction angle.

(2) $$\beta \;\cos\theta =\frac{K\lambda }{D}+4\varepsilon \sin\theta$$

where *β* is the diffraction line broadening, *K* is the form factor (0.9), *λ* is the radiation wavelength, and *ε* is the strain and *D* is crystallite size.

The UV–Raman spectra were recorded on the Fe_x_O_y_@SiO_2_/ZnO-Ag NPs by using a LABRam HR800 spectrometer (Horiba France SAS, Palaiseau, France) equipped with a CCD detector, gratings of 2400 g/mm, exciting He-Cd laser of 325 nm (Kimmon Koha Co LTD, Tokyo, Japan), and Olympus microscope objective (Olympus Corporation, Tokyo, Japan) of 40× NUV/0.47. The laser power was kept below 5 mW to prevent sample heating. The Rayleigh-scattered light was rejected with a notch filter with a window cutoff of ~300 cm^−1^, preventing recording Raman spectra below this threshold. At least three spectra were collected for each sample.

Nitrogen adsorption–desorption isotherms at 77 K were recorded on a Micromeritics ASAP 2020 analyzer (Norcross, GA, USA). The samples were degassed at 90 °C for 5 h under vacuum before analysis. Specific surface areas (SBET) were calculated according to the Brunauer–Emmett–Teller (BET) equation, using adsorption data in the relative pressure range between 0.05 and 0.30. The total pore volume (V_total_) was estimated from the amount adsorbed at the relative pressure of 0.99. The pore size distribution curves were obtained from the desorption data using the BJH (Barrett–Joyner–Halenda) model.

### 2.4. Optical Properties

To measure optical properties, the core–shell hybrid IONPs decorated with Ag were dispersed in ethyl alcohol and deposited in the form of thin films on a soda lime glass substrate, using the spin-coating technique (equipment), 2000 rpm, in three successive layers. The optical transmission spectra of the magnetic thin films were acquired at room temperature with a Perkin Elmer Lambda 35 spectrometer, operated in air, at normal incidence, in the 200–1100 nm spectral range. The absorption coefficient was calculated by equation [47]:

(3) $$\alpha =\frac{1}{d}ln\left[\frac{{\left(1-R\right)}^{2}+\sqrt{{\left(1-R\right)}^{2}+4R^{2}T^{2}}}{2T}\right]\;$$

where *d* is the film thickness, *T* is the transmittance, and *R* reflectance of the nanostructured thin films. The optical band gap energy, *E_g_*, was estimated from the fundamental absorption edge of the nanostructured thin films. Assuming allowed direct transition between valence and conduction band, the *E_g_* values for the nanostructured thin films were determined from dependence of absorption coefficient, α, on the photon energy, *hν*, by the following equation [48,49,50]:

(4) $${\left(\alpha h\nu \right)}^{2}=A\left(h\nu -E_{g}\right)$$

where *A* is a parameter that depends on the transition probability. Thus, the intercept of the extrapolation of the linear part of (*αhν*)^2^ = *f*(*hν*) curves to (*αhν*)^2^ = 0 absorption with photon energy axis is taken as the *E_g_* value.

The percentage of the adsorption efficiency, *η*, was calculated with the following equation [50]:

(5) $$\eta =\frac{C_{0}-C_{t}}{C_{0}}\times 100\%$$

where *C*_0_ and *C_t_* are the initial concentration of MB before adsorption and at time t of the sorption process.

### 2.5. Magnetic Properties

Magnetic properties were assessed at room temperature on Lake Shore’s fully integrated Vibrating Sample Magnetometer system 7404 (VSM) (Westerville, OH, USA).

### 2.6. Photocatalytic Activity

The photocatalytic properties of the synthesized samples (40 mg) were conducted by UV–Vis spectroscopic monitoring (SPECORD 210 PLUS Double-beam Spectrophotometer from Analytik Jena, Jena, Germany, equipped with a WinASPECT PLUS Software Version: 4.3.0.0) of the degradation of methylene blue (MB) dye solution (25 mL, 5 mg/L) under UV irradiation (254 nm), using a mercury UV lamp at 100 KW. The desorption test was carried out in deionized water with powders separated from MB solution after 2 h exposure to UV light and 2 days rest in demi-darkness. The resulting solutions were measured for recovered MB by optical absorption in the range of 500–750 nm.

### 2.7. Antimicrobial Activity

The antimicrobial behavior of the Ag-decorated core–shell samples was studied by diffusometry method against four standard microorganisms, three bacteria: one Gram-positive–base quality control *Staphylococcus aureus* ATCC 25923 (*S. aureus*), two Gram-negative–base quality control *Escherichia coli* ATCC 25922 (*E. coli*), and base quality control *Pseudomonas aeruginosa* ATCC 27853 (*Ps. aeruginosa*), as well as a fungus: base quality control *Candida albicans* ATCC 10231 (*Candida*). The strains used came from Thermo Scientific™ Quanti-Cult Plus™ UK Ltd., Leicestershire, and are specific, reproducible strains with a standard number of viable microorganisms, derived from authentic ATCC^®^ cultures, in the ready-to-rehydrate vial. These strains are ISO certified, cGMP (current good manufacturing practices) compliant, FDA registered, and ATCC^®^ Licensed Derivative Program member [51]. Sensitivity testing was determined using Kirby-Bauer diffusometry on Mueller Hinton standardized culture medium for bacteria and Sabouraud for fungus. The microbes were kept at 2–8 °C in a gel vial cap and then rehydrated. A bacterial inoculum was made by suspending 3–5 colonies in physiological serum. Turbidity of 0.5 was obtained on the Mc Farland scale, measured with a nephelometer (for fungus, 0.5 McFarland = 1 × 10^6^ to 5 × 10^6^ cells per mL; for bacteria, 0.5 McFarland = 1.5 × 10^8^ cells per mL). A buffer was inserted into the calibrated suspension and seeded by rotating the plate by 60 degrees, obtaining uniform seeding. A set of control samples were carried out as follows: on the surface of the inoculated medium: 1 blank 6 mm control disc (negative control), a disc with Ciprofloxacin 5 mcg (positive control for bacteria), and a disc with fluconazole 25 mcg (positive control for fungus). The set of samples with study substances was placed on other plates, next to a blank disc. The study substances were prepared as follows: 10 μL dispersions of 2 mg MNPs into 2.5 mL dimethyl sulfoxide (DMSO) were deposed on a filter paper disc of 6 mm diameter and placed on the culture medium plate. After 15 min. of depositing the discs, the plates were placed in the thermostat at 35 ± 2 °C, in aerobiosis, for 18–24 h. The diameters, in millimeters, of the inhibition areas (no bacterial growth around the discs soaked with the studied substances) were read with the help of a graduated line [15].

## 3. Results and Discussion

### 3.1. Morphology and Structure

**Figure 1:**
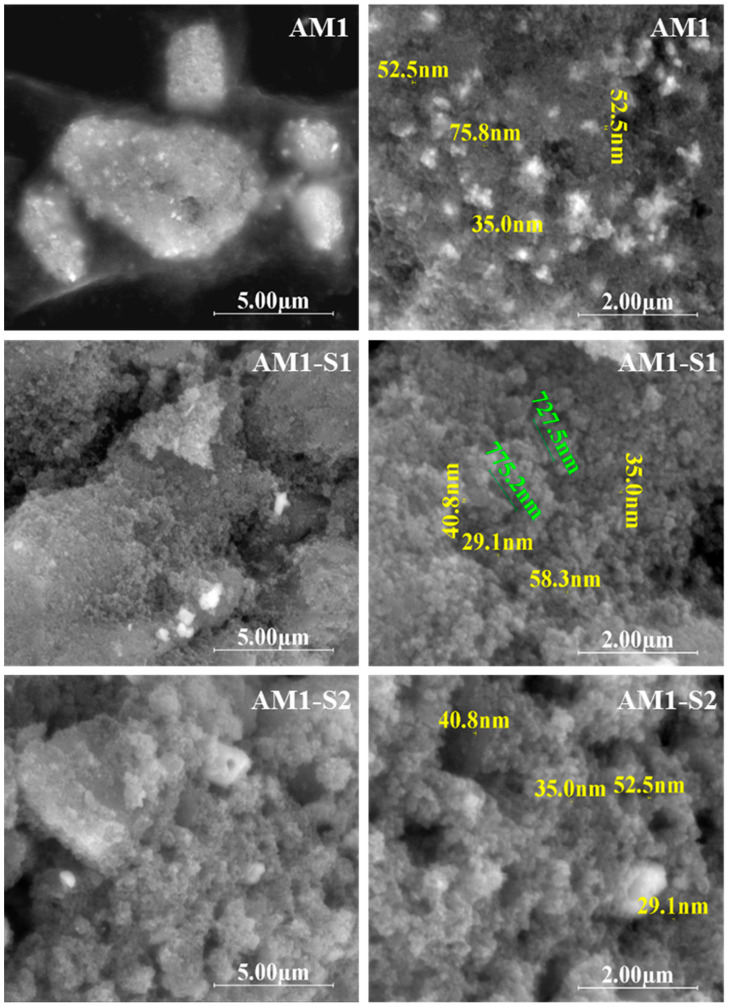

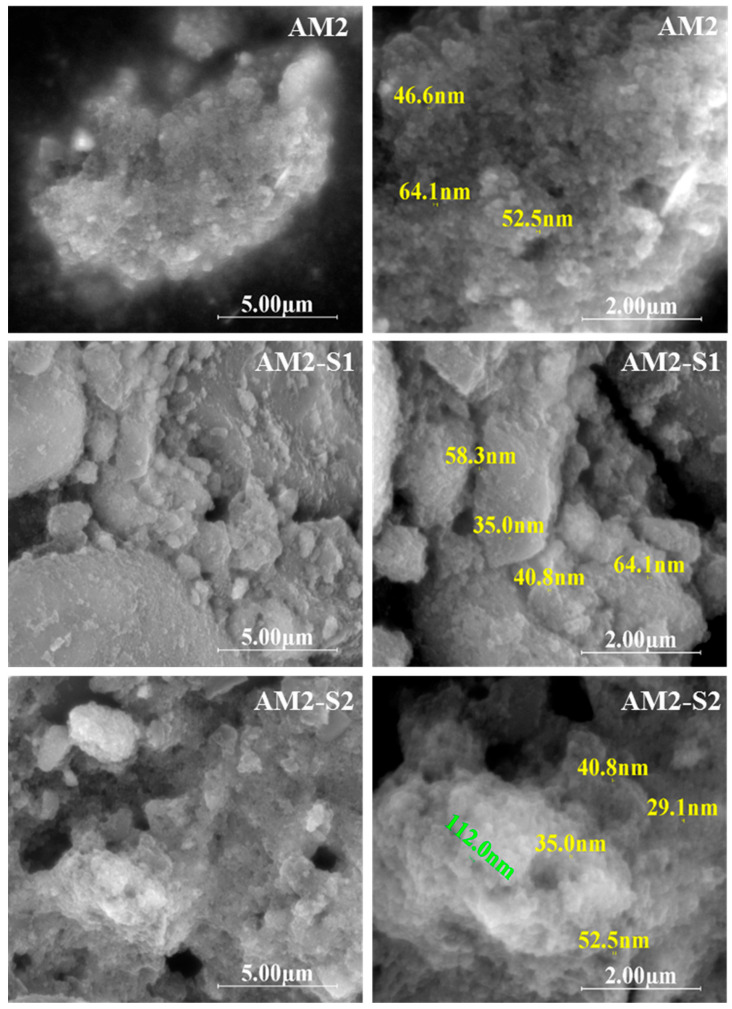
Top-view SEM images of Ag-decorated IONPs (AM1 and AM2) and the corresponding core–shell composites nanoparticles (AM1-S1/2 and AM2-S1/2).

The top-view SEM micrographs in Figure 1 show a decrease in the size of the core–shell nanoparticles, compared to the corresponding core-type nanoparticles. In the case of MNPs synthesized at 95 °C, the decrease was from 35–70 nm (AM1) to 29–50 nm (AM1-S1/2), and for the samples obtained at 80 °C, the variation was from 46–75 nm (AM2) to 35–60 nm (AM2-S1/2).

The biggest nanoparticle size indicates the agglomeration of the silver-decorated AM2 core, in contrast with the corresponding silver-free M2 sample (24 nm) [4]. Strings of self-assembled superparamagnetic nanoparticles with an approximate length of 700–900 nm can be observed for sample AM1-S1 (Figure 1-dimensions in green). Additionally, a complex porous morphology is depicted for all the Ag-decorated samples, especially the core–shell IOMNPs (Figure 1). Based on the radial dimension, three classes of pores are considered by IUPAC, namely micropores (r ≤ 2 nm), mesopores (2 nm < r < 50 nm), and macropores (r > 50 nm) [52].

Figure S1 corresponds to hierarchically structured porous materials [52]. While the Ag-Fe_3_O_4_ morphology includes a meso (35–90 nm)–macro (120–200 nm) pores bimodal distribution, and the one for the core–shell Ag-Fe_3_O_4_@SiO_2_ sample has a trimodal pore system of meso (35–99 nm)–macro (110–200 nm)–macro (300–800 nm) pores randomly distributed. We consider that the bimodal distribution in the case of Ag-Fe_3_O_4_ is generated by the self-assembly of these superparamagnetic quantum dots nanoparticles (mesopores) and, respectively, by the surfactant effect of citrate used as a reducing agent in Ag decoration (macropores). Regarding the trimodal porosity of the Ag-Fe_3_O_4_**@**SiO_2_ samples, it is generated by the templating agent-free sol-gel synthesis of the SiO_2_ coating, superimposed with the surfactant effect of citrate. The SEM-EDX spectra and maps of the investigated samples confirmed the presence of silver decoration and the silica shell onto M1-S1/2 and M2-S1/2 IOMNPs particles (Figures S2 and S3).

**Figure 2:**
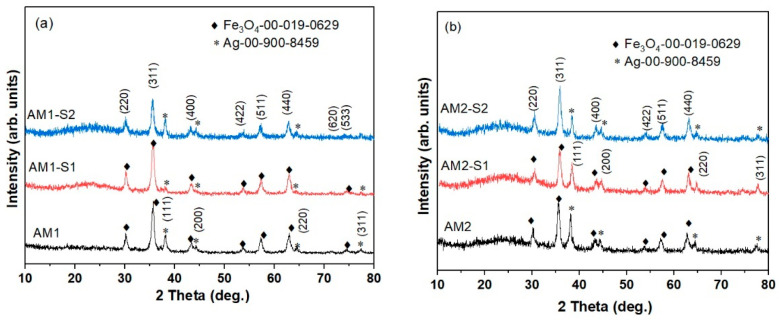
XRD patterns of the (**a**) AM1(-/S1/S2) and (**b**) AM2(-/S1/S2) samples.

The XRD patterns of AM(1/2)-S(1/2) and AM1-S1 samples (Figure 2) exhibit the diffraction lines corresponding to magnetite (Fe_3_O_4_) with cubic symmetry (JCPDS no. 00-019-0629) and Ag (JCPDS no. 00-900-8459) [53,54]. This is dissimilar to the corresponding Ag-free iron oxide cores, where a mixture of Fe_3_O_4_ and γ-Fe_2_O_3_ was depicted [4].

The lattice parameters and crystallite size of Fe_3_O_4_ calculated from the XRD data are listed in Table 1. The crystallite size, estimated using Scherrer’s equation for the (311) plane, was around 13 nm for the AM1 series and 15 nm for the AM2 series. For comparison, the crystallite size was calculated using the WH method. The results showed that the crystallite sizes are slightly different from those estimated using Scherrer’s equation, indicating that the broadening of the diffraction lines in the samples is not only due to smaller crystallite size, but also due to strain [46]. A broad diffraction line can be observed at 23.15° for the core–shell composite samples corresponding to the amorphous SiO_2_ shell. The almost double silver content was encountered for the low-temperature processed Fe_3_O_4_ core (80 °C) in AM2, in contrast with the one obtained at 95 °C (AM1). The lower silver content in the core–shell NPs is very likely due to the low affinity of the noble metal NPs for silica, as reported in the literature [55].

**Figure 3:**
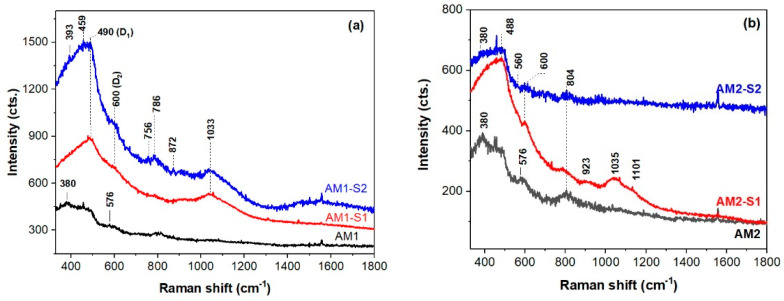
UV–Raman spectra of the (**a**) AM1(-/S1/S2) and (**b**) AM2(-/S1/S2) samples.

The UV–Raman spectra of the investigated samples are shown in Figure 3, except for magnetite and hematite, which are strong Raman scatterers, and singles and mixtures of iron oxides and oxy-hydroxides are difficult to observe by Raman spectroscopy, due to weak and overlapped bands [56,57]. Although the Raman band at about 380 cm^−1^ of the AM1 and AM2-(-/S2) (Figure 3) can belong to iron oxy-hydroxides [58], the A1(TO) modes of the Ag-doped ZnO NPs were reported to have a similar band position [59]. Considering the successful shielding effect of the SiO_2_ coat for the Fe_3_O_4_ core, the latter band originates from ZnO in the case of the hybrid shell SiO_2_/ZnO of the AM2-S2 NPs (Figure 3b). The spectral features at 380 and 570 cm^−1^ (A1 (LO) modes, due to oxygen vacancies and interstitial zinc [60]) of the AM2-S2 nanoresonators (Figure 3b), widen and are less intense, which will not interfere with SERS detection of pollutants and in the theragnostic [35,36]. The Fe-O vibrations [58] at 576 cm^−1^ are present in the AM(1/2) spectra in Figure 3. One of the conditions to obtain a SERS effect, due to silver plasmonic nanostructures, is that Ag-decorated Fe_3_O_4_@SiO_2_/ZnO substrate gives a weak Raman background under a certain wavelength laser excitation [61]. The weakest UV–Raman signal of the investigated samples was recorded for the AM2-S2 sample. Moreover, immobilized and aggregated silver nanostructures were reported [61] to trigger Raman signal enhancement of substance traces.

UV–Raman spectroscopy is very sensitive to the surface of the SiO_2_ cover in the core–shell structures [62]. Thus, Raman bands at ~490, 600, 790, 923, 1035, and 1101 cm^−1^ (defect modes D1 and D2 of the 4- and 3-membered cycloxyloxane rings, stretching Si-O modes [62]) in the AM(1/2)-S1 and AM1-S2 spectra are typical of the silica shell. This is analogous with the SiO_2_ shells of the corresponding Ag-fee Fe_x_O_y_/SiO_2_ NPs, i.e., M(1/2)-S1 NPs, reported elsewhere (Fe_x_O_y_ stood for a mixture of Fe_3_O_4_ and γ-Fe_2_O_3_) [4]. The E_2_^high^ modes of the hexagonal ZnO at ~440 cm^−1^ [59] are very likely masked by the Si-O vibration modes.

### 3.2. Textural Analysis

**Figure 4:**
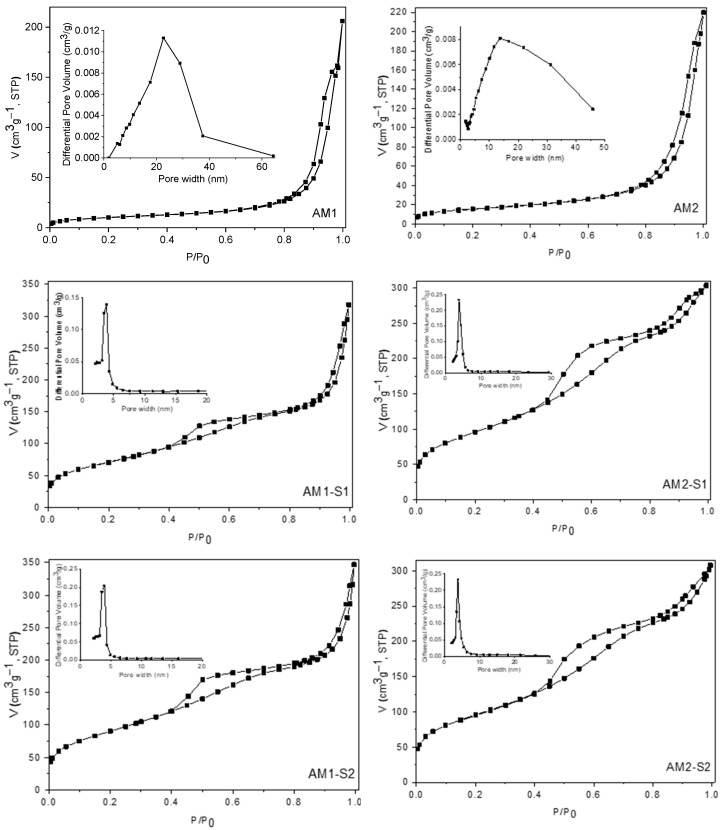
N_2_ adsorption–desorption isotherms and pore size distributions (inset of the figures) of the investigated samples.

The N_2_ physisorption measurements were carried out to investigate the textural features of the samples under discussion. Thus, the nitrogen adsorption–desorption isotherms and the corresponding pore-size distribution (PSD) curves of the samples are shown in Figure 4. All the isotherms are of type IV, according to the IUPAC classification [63,64]. The values of BET surface areas and the total pore volumes are listed in Table 1. The AM1 and AM2 samples display the isotherms typical for materials with large mesopores having steep condensation under a very high relative pressure (p/p_0_ > 0.8). This type of porosity is given mainly by the interparticle voids. For the samples containing silica, the capillary condensation phenomena appear at relative pressures higher than 0.4, giving rise to hysteresis loops of H3 type, which could be an indication for the presence of slit-shape pores [65,66]. The PSD graphs confirm the presence of small and uniformly distributed mesopores ranging from 3 to ~6 nm, with slightly smaller values for the AM2-S1 and AM2-S2 samples.

The high total pore volumes determined for all samples could have a beneficial influence on their photocatalytic properties. Comparing the SBET values of these samples containing Ag nanoparticles deposited on their surfaces with those of the corresponding samples without Ag (data not shown) [4], a slight decrease in all values was observed, as expected, taking into account that the deposition of Ag nanoparticles occurs on the entire surface (internal and external) of the materials, a part of the pores being clogged during this process.

### 3.3. Magnetic Properties

**Figure 5:**
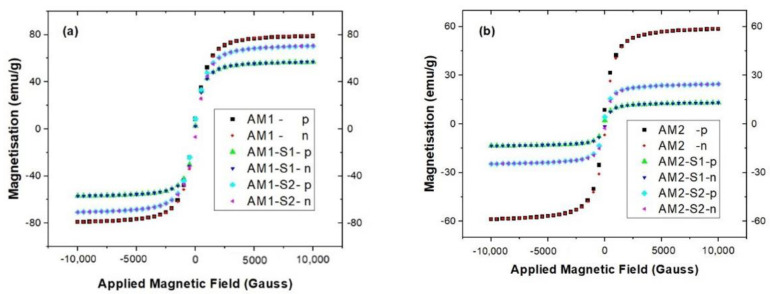
Magnetic hysteresis loops of the in (**a**) AM1(-/S1/S2) and (**b**) AM2(-/S1/S2) samples.

The magnetization hysteresis curves in Figure 5 and almost no remanence and coercivity (Table 1) for the Ag-decorated iron oxide samples (AM1 and AM2), together with the corresponding Ag-decorated core-hybrid shell of SiO_2_/ZnO modified (M1-S1/2 and M2-S1/2) IOMNPs indicate that all samples are superparamagnetic. As expected, higher saturation magnetization, Ms, calculated with the Langevin function was recorded for the higher temperature (95 °C) obtained AM1 (see Table 1). In addition, the Ms value of 83.23 emu/g for the AM1 is bigger than the one for the Ag-free sample M1 (70.12 emu/g) [4], being closer to the 90 emu/g the bulk Fe_3_O_4_ [67]. The same trend was observed for the Ms values of the AM1 set (AM1-(-/S1/S2), in contrast with the Ag-free M1 set of samples [4]. This finding is in line with the findings for the Ag/Fe_3_O_4_ NPs, where a smaller crystal domain size was observed as dopant content increased or there was richer Fe^2+^ content in the cubic inverse spinel structure of the Fe_3_O_4_ cores [68].

Smaller Ms values were obtained for the Ag-decorated NPs with nonmagnetic SiO_2_ and hybrid SiO_2_/ZnO shells, very likely due to decreasing subsequences in magnetism and quenching of surface magnetic moments [69]. While the smallest Ms value was measured for the AM(1/2)-S1 samples with SiO_2_ shell, the hybrid core–shell samples, AM(1/2)-S2, encountered intermediate values (Table 1). Although a 60% decrease of the Ms values was recorded for successive covering of the Fe_3_O_4_ core with materials without magnetic response [70], only an 8.86% decrease was noticed in the case of the ternary AM1-S2 sample and 77.87% for the AM2-S2, respectively.

Negligible magnetic remnants and coercivity imply a superparamagnetic behavior of all the samples studied in this work. The Ms values exceeding 20 emu/g (except for the AM2-S1 sample) enable the recovery of all the samples presented here by using an external magnetic field.

### 3.4. Optical Properties

**Figure 6:**
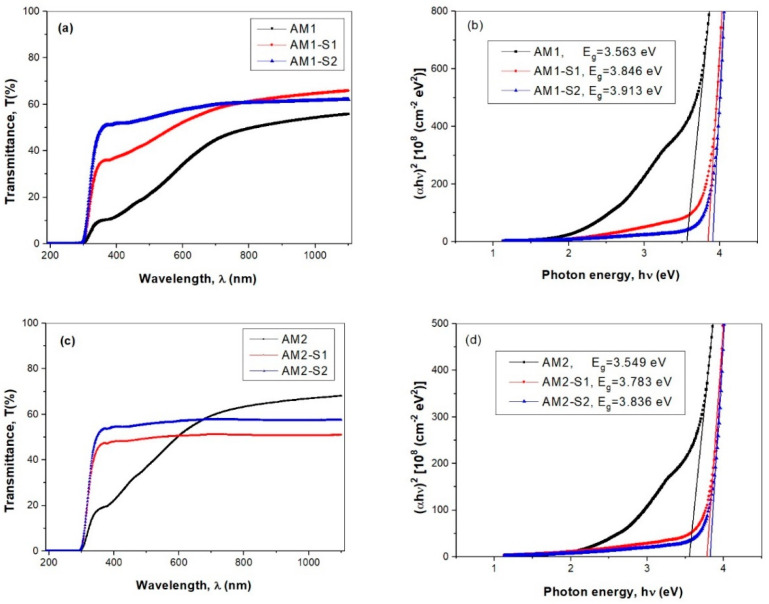
Optical transmittance spectra (**a**,**c**) and Tauc plots (**b**,**d**) of the simple iron oxide (M1 and M2), together with the corresponding core–shell silica modified (M1-S1/2 and M2-S1/2) thin films.

Since the optical band gap energy (E_g_) helps predict the photochemical properties of semiconductors [45,71], the optical transmittance spectra and calculated E_g_ values of the silver-decorated iron oxide, and the corresponding core–shell silica-modified thin films are illustrated in Figure 6. The E_g_ values have been determined by extrapolating the linear parts to zero absorption with the photon energy axis. The intercepts of these extrapolations, taken as the value of the optical band gap energy, E_g_, are presented in Figure 6b,d. A significant blue-shift was observed for the Ag-decorated core–shell NPs up to 600–700 nm spectral range the optical transmission percentage decreases in succession: AM(1/2)-S2 > AM(1/2)-S1 > AM(1/2) (Figure 6a,c), with increasing silver content. The Ag films were reported to have high absorption over the short wavelength domain and, hence, lower optical transmittance [72]. The optical spectra in Figure 6a show improved transmittance of the core–shell-containing films in the AM1 set, in comparison with corresponding the AM1 core. Conversely, the highest percentage of transmission is recorded for the AM2 core film (68%), within the 684–1200 nm (NIR) range (Figure 6c).

A significant blue shift was observed for the Ag-decorated core–shell samples, especially for those with ZnO-doped silica shells (AM1/2-S2 samples in Figure 6b,d). This finding is confirmed by a slight increase of the E_g_ values, from those of the Ag-decorated cores (AM1 and AM2) to core–shell samples, e.g., AM(1/2)-S(1/2) (see Figure 6b). The E_g_ values of the Ag-decorated cores are similar to the ones reported for the Ag-free Fe_3_O_4_ NPs (~3.7 eV) [73].

### 3.5. Antimicrobial Activities

**Figure 7:**
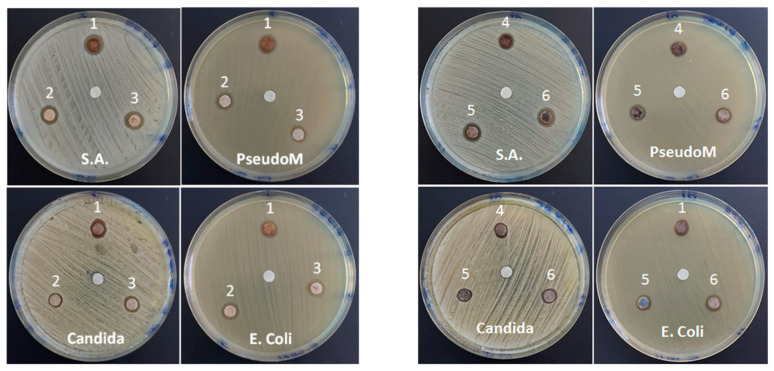
Disk diffusion assay for the investigated samples: AM1(1), AM1-S1(2), AM1-S2(3), AM2 (4), AM2-S1(5), and AM2-S2(6).

The samples show antimicrobial and anti-fungal activity, with values of the measured inhibition zone ranging between 9.5 and 10.7 mm. As can be seen from Figure 7, the most sensitive bacterium to the newly synthesized samples is the gram-positive one (*S. aureus*). At the same time, the two AM(1/2)-S2 samples with similar Ag content (~7%), hybrid SiO_2_/ZnO shell and large surface area of 328.8 and 342.5 m^2^g^−1^ (see Table 1), respectively, seem to be more active than those with single a SiO_2_ shell, AM1/2-S1, and core samples of AM(1/2), both for bacteria and fungus, meaning the biocide activity of the silver nanoparticles [74], AgNPs, is completed by the ZnO component. Both Ag^+^ and Zn^2+^ are known to exert bactericidal behavior by the reactive oxygen species (ROS) production [17]. This is analogous to Matusoiu et al.’s findings [75] for the sol-gel obtained SiO_2_@Fe_x_O_y_@ZnO materials, where antimicrobial activity was ruled by ZnO content (minimum inhibitory concentration of 1 mg/mL), and ROS were generated by ZnO. Furthermore, electrostatic interactions are expected between Ag Nps and microorganisms [76] in the Ag-decorated samples.

Moreover, the positive control test to assess the antimicrobial efficiency of the studied MNPs, in addition to the negative control test with a blank disc, without active substance (present in all the images in Figure 7), performed with ciprofloxacin and fluconazole for bacteria and fungus, respectively, are illustrated in Figure S4. The differences between the inhibition zones observed in the mentioned figures cannot be directly correlated with the antibacterial/antifungal efficiency of the studied MNPs, based on the different amounts of medicine (5 and 24 mcg) and MNPs (8 mcg) deposited on the disk. The values for the diameter of the minimally active inhibition zones are specific to each medicine and are standardized/validated based also on clinical studies. In the case of this study, since we are talking about new compounds, it is very relevant that clear inhibition zones were highlighted for quantities comparable to or lower than those of the standardized drugs in the positive control test. This represents a very good stage for optimizing the properties of the studied materials. Since the contact of the bacteria and dissolved control antibiotics at the molecular level is much more effective, the basic requirement for increasing the activity of MNPs is to decrease the size of the nanoparticles, in order to increase the specific surface in direct contact with the bacteria.

### 3.6. Nanosorption and Photocatalytic Activities

**Figure 8:**
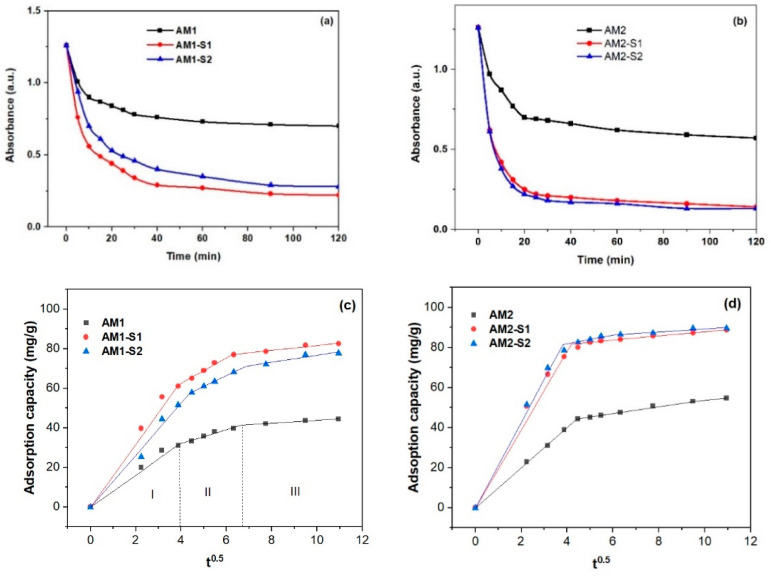
Dark adsorption (**a**,**b**) and adsorption capacity of MB on AM(1/2)-S(1/2) NPs vs. $\sqrt{t}$ (**c**,**d**) (c(MB) = 5 mg/L, V = 25 mL of MB solution and w(AM(1/2)-S(1/2) = 0.040 g at room temperature).

Quick adsorption of the MB (<30 min.) on the Ag-decorated ternary core–shell samples (Figure 8 and Figure S4) was followed by a slower adsorption rate up to equilibrium (120 min). It is very likely that, in the second, slower stage, fewer active sites were available on the surface, so MB was adsorbed on the internal surface (pores). After two hours, the percentage of adsorption capacity (q_t_) in Figure 8c,d varied with the S_BET_ values in Table 1. Thus, the biggest adsorption capacity/efficiency (~90 mg/g) and S_BET_ value of 344.6 m^2^/g were recorded for the AM2-S1 NPs, and vice versa, the lowest η of ~44 mg/g corresponded to the lowest S_BET_ of 36 m^2^/g in case of the AM1 NPs. Analogous with the corresponding Ag-free NPs [4], the Ag-decorated NPS with single SiO_2_ and hybrid SiO_2_/ZnO shells had the biggest absorption efficiency and an early steeper sorption process, in comparison with the core AM1 and AM2 NPs. Optimization of the MB sorption process requires additional kinetic information on the mechanism of the sorbent–sorbate interaction [77].

Usually, the adsorption process is limited by intraparticle diffusion. Since the unconstrained linear fitting of the adsorption capacity versus $\sqrt{t}$ (Figure 8c,d) by an intraparticle diffusion model does not pass through the origin for the two-hour adsorption process, the intraparticle diffusion model is not the only rate-limiting step. The constrained linear fit through the origin of the 2 h adsorption data in Figure S5 gives smaller K_id_ and R^2^ values (Table S1), which confirms the multi-step MB adsorption process. Three steps were used for MB adsorption on the Fe_3_O_4_@SiO_2_/Zn-Ag NPs, with iron oxide core obtained at 95 °C. Typically, the first step is assigned to external diffusion, while the second and third steps belong to intraparticle diffusion in micropores and mesopores, as well as adsorption/desorption on and from the active sites [78]. In the case of the AM2 set of NPs, even two stages of adsorption seem appropriate. For the first stage, the R^2^ value is close to 1 for the linear fit of the adsorption capacity versus $\sqrt{t}$ (Figure 8c,d and Table 2), analogous to the one corresponding to the Ag-free M(1/2)-S(1/2) NPs [4].

If the k_id_ values for nanoparticles whose cores were obtained at the higher temperature of 95 °C are similar for the M1 and AM1 sets, two times bigger values were recorded for the AM2 set, in comparison with the M2 set. As the intercept of the linear fit in steps 2 and 3 increases for the core–shell NPs (see Table 2), the boundary layer thickness has a greater influence on the adsorption process.

**Figure 9:**
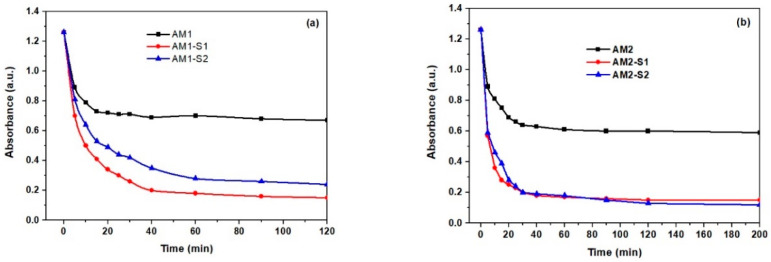

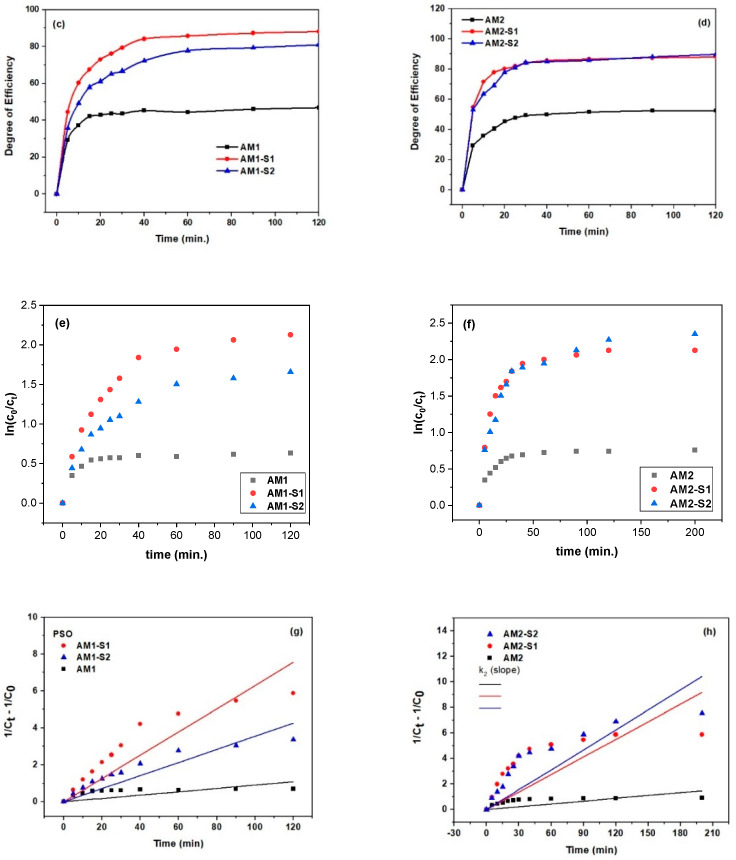
Photocatalytic degradation of MB on Ag-decorated AM1, AM2, and AM(1/2)-S(1/2) nanocatalysts via UV–Vis spectrophotometry (**a**,**b**), MB discoloration efficiency (**c**,**d**), photodegradation kinetics: PFO (**e**,**f**) and PSO (**g**,**h**) (c(MB) = 5 mg/L, V = 25 mL of MB solution and w(AM(1/2)-S(1/2) = 0.040 g at room temperature).

The calculated percent of MB photodegradation versus irradiation time is plotted in Figure 9c,d (Equation (4)). As expected, MB itself undergoes a specific chemical degradation under the action of UV–Vis light. In this study, we observed the degradation of 4% MB after 120 min. irradiation at 254 nm (Figure S6). This absorbance decrease was subtracted for the subsequent MNPs photocatalysis investigation.

The MB degradation mechanism and by-product formation in the presence of various photocatalysts were studied and reported in detail in scientific literature. Typically, during a photocatalytic process, a semiconductor material turns into a catalyst that is activated by the absorption of photons and that generates reactive species with oxidizing character. Thus, electron-hole pairs (é-h^+^, i.e., excitons) are generated on the photocatalyst surface, so that free electrons generate superoxide ^·^O^2−^ radicals by reducing O_2_ molecules from the air, while the h^+^ oxidizes water molecules with the formation of ·OH radicals generate hydrogen peroxide (H_2_O_2_). Both ^·^O_2_^−^ and H_2_O_2_ are highly oxidizing species and very efficient in the degradation of organic compounds through redox reactions [79]. Based on gas and liquid chromatography coupled with mass spectroscopy (GC-MS, LC-MS) and FTIR spectra, it was highlighted that the oxidative degradation of MB under the influence of radicals formed in the presence of light radiated catalysts proceeds in the following steps [79]:

1. Formation of the MB de-methylated intermediates, called Azure A, Azure B, and thionine acetate, with the preservation of the chromophore poly-heterocyclic group and the release of Cl^−^ (most ionized, in the independent state).
2. Breaking the poly-heterocyclic linkages into central, followed by side, aromatic rings, with the formation of fragments and possible residual single-ring structure products.
3. Degradation of resulting fragments into various intermediate species (aniline, phenol, carboxylate species, R-NH_3_^+^, etc.).
4. Degradation of intermediate species, until final products, which are volatile low molecular weight and less toxic products (CO_2_, H_2_O, SO_4_^2−^, and NO_3_^−^).

According to this mechanism, a similar behavior of our samples seems to indicate that, during the discoloration of the solution, associated with the decrease of the 665 nm peak intensity, following the degradation of the MB chromophore group, no other intermediates with chromophore groups are formed. To obtain kinetic information on the MB photodegradation, two types of plots (Figure 9e–h) were used, namely ln(c_o_/c_t_) versus time for pseudo-first-order (PFO) kinetics and (1/c_0_–1/c_t_) versus t^0.5^ for pseudo-second-order (PSO) kinetics [80] (see Table 3). Linear fit of the data in Figure 9g and h for a shorter time was presented in Table S2.

By comparison with the Fe_3_O_4_@ZnO [62] and Fe_3_O_4_@Ag@TiO2 [80] nanocatalysts (Table 4), slightly improved photocatalytic performances, regarding MB discoloration for 2 h, were obtained in the case of Ag-Fe_3_O_4_@SiO_2_/ZnO, AM2-S2.

Analogous to the multilayer Fe_3_O_4_@SiO_2_@ZnO–Ag microspheres used for rhodamine degradation [84], reduced recombination of the photogenerated electron/hole is the plausible explanation of the enhanced photocatalytic degradation of the MB on the surface of the Ag-decorated Fe_3_O_4_@SiO_2_/ZnO. As expected, the role of the Ag NPs was to trap electrons [85] and, hence, to slow the electron/hole recombination.

## 4. Conclusions

The Ag-decorated ternary Fe_3_O_4_@SiO_2_/ZnO with the hierarchically interconnected porous structure were successfully obtained by a three-step process (coprecipitation of the iron oxide core, sol-gel deposition of the shells, and citrate-based step of the silver decoration) for photocatalytic and antimicrobial purposes. A single Fe_3_O_4_ phase was depicted in the core of the Ag-decorated ternary Fe_3_O_4_@SiO_2_/ZnO NPs, in contrast to the Ag-free counterparts, composed of a mixture of the Fe_3_O_4_ and γ-Fe_2_O_3_ phases.

The saturation magnetization (Ms) of the Fe_3_O_4_ cores enhanced for silver decorated Fe_3_O_4_@SiO_2_/ZnO superparamagnetic nanoparticles (79.34 emu/g) synthesized at 95 °C, in contrast to the Ag-free counterparts (70.12 emu/g), under the influence of the reducing agent (citrate), also acting as a surfactant. Thus, for the core–shell Ag decorated samples, the pore volume increased by moving to a trimodal network of interconnected pores, with diameters ranging from the mesoscale (below 100 nm) to macropores with diameters up to 800 nm.

Improved adsorption and photocatalytic activity were noticed for the Ag-decorated photocatalysts with a hybrid SiO_2_/ZnO shell, very likely due to reduced recombination of excitons, due to the silver, presence of ZnO in the hybrid shell, and bigger specific surface.

The Ag-decorated core-hybrid shell Fe_3_O_4_ exhibited excellent antimicrobial activity against *S. aureus* and good restricting growth of the gram-negative strains, such as *Ps. aeruginosa*, *E. coli* and antifungal activity against *Candida*. The antifungal activity of these core–shell MNPs seems to be close to the one of fluconazole.

Multifunctional nanomaterials combining superparamagnetic Fe_3_O_4_ core and plasmonic properties of metallic silver nanoparticles are very attractive for reusable surface-enhanced Raman spectroscopy (SERS) purposes. Hence, SERS activity of the Ag-decorated Fe_3_O_4_@SiO_2_/ZnO will be the subject of further studies.

## Figures and Tables

**Table 1:** Structural and physical parameters of investigated MNPs.

Sample	XRD Results							Textural Parameters		Magnetic Parameters **		
	Phases	wt%	2θ (°) (311)	FWHM (°)	Lattice Parameter a = b = c (Å)	Crystallite Size (311) (Å)	Crystallite Size (WH) (Å)	S_BET_ (m^2^g^−1^)	Pore Volume (cm^3^g^−1^)	Ms [emu/g]	*μ/k_B_* [K/Oe]	Quality Factor
AM1	Fe_3_O_4_	91.2	35.619	0.627	8.357	139	117	36.0	0.318	83.23	0.73	6.52 × 10^−4^
	Ag	8.8	-	-	-	-	-					
AM1-S1	Fe_3_O_4_	97.7	35.601	0.647	8.349	135	103	258.0	0.491	58.47	1.14	8.03 × 10^−5^
	Ag	2.3	-	-	-	-	-					
AM1-S2	Fe_3_O_4_	93	35.480	0.590	8.352	148	110	328.8	0.536	73.85	0.78	6.10 × 10^−4^
	Ag	7	-	-	-	-	-					
AM2	Fe_3_O_4_	81	35.507	0.609	8.368	143	126	55.7	0.290	60.57	0.947	6.80 × 10^−4^
	Ag	19	-	-	-	-	-					
AM2-S1	Fe_3_O_4_	84	35.473	0.624	8.371	140	119	344.6	0.471	13.41	1.29	9.52 × 10^−4^
	Ag	16	-	-	-	-	-					
AM2-S2	Fe_3_O_4_	92.6	35.481	0.588	8.387	148	127	342.5	0.475	24.87	1.29	7.20 × 10^−4^
	Ag	7.4	-	-	-	-	-					
Reference (00-019-0629)	-	-	35.42	-	8.396	-		$**M\left(T,H\right)=M_{S}\left[\coth\left(\frac{\mu H}{k_{B}T}\right)-\frac{k_{B}T}{\mu H}\right]$ T[K] is temperature, H[Oe] is intensity of the applied field and [Oe] stands for [Gauss]				

**Table 2:** Intraparticle diffusion kinetic parameters (q_t_ = k_id_t^0.5^ +C) of MB adsorption onto AM(1/2)-S(1/2) NPs (linear fit).

Sample	AM1	AM1-S1	AM1-S2	AM2	AM2-S1	AM2-S2
k_id_ (I) (mg/g h)	8.0733 ± 0.31805	15.84282 ± 0.63744	13.12463 ± 0.35639	9.9987 ± 0.10022	19.49776 ± 0.79502	21.35053 ± 0.64258
K_id_ (II) (mg/g h)	3.44207 ± 0.56132	6.45414 ± 0.62699	5.52582 ± 0.10893	1.72125 ± 0.0643	1.16797 ± 0.18842	2.15124 ± 0.43609
C_II_(mg g^−1^)	18.39	36.70	33.30	36.67	76.79	73.28
K_id_ (III) (mg/g h)	0.7473 ± 0.10607	1.25483 ± 0.36674	1.76237 ± 2.7× 10^-5^	1.24082 ± 0.0818	0.98705 ± 0.04854	0.76131 ± 0.17163
C_III_(mg g^−1^)	36.36	69.16	59.10	41.25	78.02	81.73
Break point (min^0.5^)	3.97	3.909	4.38	4.43	4.18	3.81
	6.66	6.24	6.85	9.51	6.82	6.08
R^2^ (I)	0.99383	0.99357	0.99706	0.9997	0.9965	0.99729
R^2^ (II)	0.9495	0.98148	0.9992	0.99722	0.97464	0.92406
R^2^ (III)	0.98025	0.9213	0.9999	0.9956	0.99759	0.90773

**Table 3:** Kinetical parameters for the 120 min. photocatalysis process of MB degradation under UV irradiation and kinetical models [81].

Sample	K_0_ (min^−1^)	R^2^	K_1_ (min^−1^)	R^2^	K_2_ (min^−1^)	R^2^
AM1	0.00622 ± 0.00146	0.64429	0.00827 ± 0.00187	0.66282	0.009 ± 0.00194	0.68356
AM1-S1	0.01152 ± 0.00243	0.6925	0.02569 ± 0.00405	0.80105	0.06298 ± 0.0597	0.91747
AM1-S2	0.01028 ± 0.002	0.7268	0.01944 ± 0.00285	0.82341	0.0355 ± 0.0322	0.92405
AM2	0.00693 ± 0.0015	0.68064	0.00966 ± 0.00195	0.71114	0.01115 ± 0.00207	0.74435
AM2-S1	0.0118 ± 0.00276	0.6467	0.02697 ± 0.00521	0.72787	0.06796 ± 0.00959	0.83391
AM2-S2	0.01177 ± 0.00259	0.67295	0.02718 ± 0.00458	0.77922	0.07112 ± 0.00725	0.90586
*Equation* [81]	*C_t_*/*C*_0_ *=* 1 − (*k*_0_/*C*_0_)*t*		*ln*(*C_t_*/*C*_0_) *= k*_1_*t*		(1/*C_t_* − 1/*C*_0_) = *k*_2_*t*	
Linear fit of data in Figure	Figure 9c,d		Figure 9e,f		Figure 9g,h	

**Table 4:** Photocatalysis performances of some nanocatalysts.

Sample	MB Photodegradation Efficiency (%)	Time (min.)	Rate Constant, k (min^−1^)	Reference
Fe_3_O_4_	89	140	k_0_ = 0.0085	[81]
Fe_3_O_4_-SiO_2_	97	140	k_0_ = 0.102	[81]
Fe_3_O_4_@Ag@TiO_2_	79.9	120	k_1_ = 0.0120	[80]
Fe_3_O_4_@ZnO	88.5	120		[63]
Fe_3_O_4_@SiO_2_@ZnO cubes	55	90	k_1_ = 0.0074	[82]
Ag-Ag_2_O-ZnO (VIS light)	97.3	60	k_1_ = 0.057	[83]
Ag-Fe_3_O_4_@SiO_2_/ZnO	90.47	120	k_2_ = 0.07112	This work

## Data Availability

All the data supporting the findings of this study are available within the article.

## Supplementary Materials

The following supporting information can be downloaded at: https://www.mdpi.com/article/10.3390/nano12244452/s1, Figure S1: SEM images with pore sizes of the Ag-decorated core–shell IOMNPs, Figure S2: SEM-EDX spectra and maps of the Ag-decorated core–shell IOMNPs particles; Figure S3: SEM-EDX mapping of the Ag-decorated core–shell IOMNPs; Figure S4: Disk diffusion assay for the control samples carried out on the surface of the inoculated medium: 1 blank 6 mm control disc (negative control), discs with ciprofloxacin 5 mcg (positive control for S.A., E. coli and Pseudomonas bacteria) and fluconazole 25 mcg (positive control for Candida fungus); Figure S5: Variation of the intensity of main peak optical absorption of the MB solution after 120 min under UV irradiation (a) or dark (b) without NPs (MB) and in the presence of investigated NPs samples, AM(1/2)-S(1/2); Figure S6: The intensity of the main optical absorption peak of the MB solution, before (t = 0 min) and after 120 min under UV irradiation without the presence of investigated samples, used for photocatalytic tests; Figure S7: Adsorption capacity versus $\sqrt{t}$ (min.^0.5^) and linear fit of MB degradation on the AM1 (a) and AM2 NPs (b). Table S1: Kinetic parameters (intraparticle diffusion model) of the adsorption process of MB on the surface of the nanocatalysts during 2 h; Table S2: Kinetical parameters of the photocatalytic process of MB degradation under UV irradiation within 0–30 min. range. Ref. [81] is cited in Supplementary Materials.
