# Supplementary material for: Ag-Decorated Iron Oxides-Silica Magnetic Nanocomposites with Antimicrobial and Photocatalytic Activity
Source: Nanomaterials (Basel). 2022 Dec 15;12(24):4452. doi: 10.3390/nano12244452 (PMC9783173; doi:10.3390/nano12244452)
Supplement: Supplementary file 1 [file nanomaterials-12-04452-s001.zip › nanomaterials-2079516-supplementary.pdf]

Journal: NANOMATERIALS

Paper (Article): *Ag-decorated iron oxides-silica magnetic nanocomposites with antimicrobial and photocatalytic activity*

Authors: **Viorica Muşat[1,*], Lenuța Crintea (Căpățână)[1], Elena-Maria Anghel[2,*], Nicolae Stănică[2], Irina Atkinson[2], Dana Cristina Culiţă[2], Liliana Baroiu[3], Nicolae Țigău[4], Alina Cataragiu Cioromila[5], Andreea-Veronica Botezatu (Dediu)[4], Oana Carp[2]**

Supplementary Information

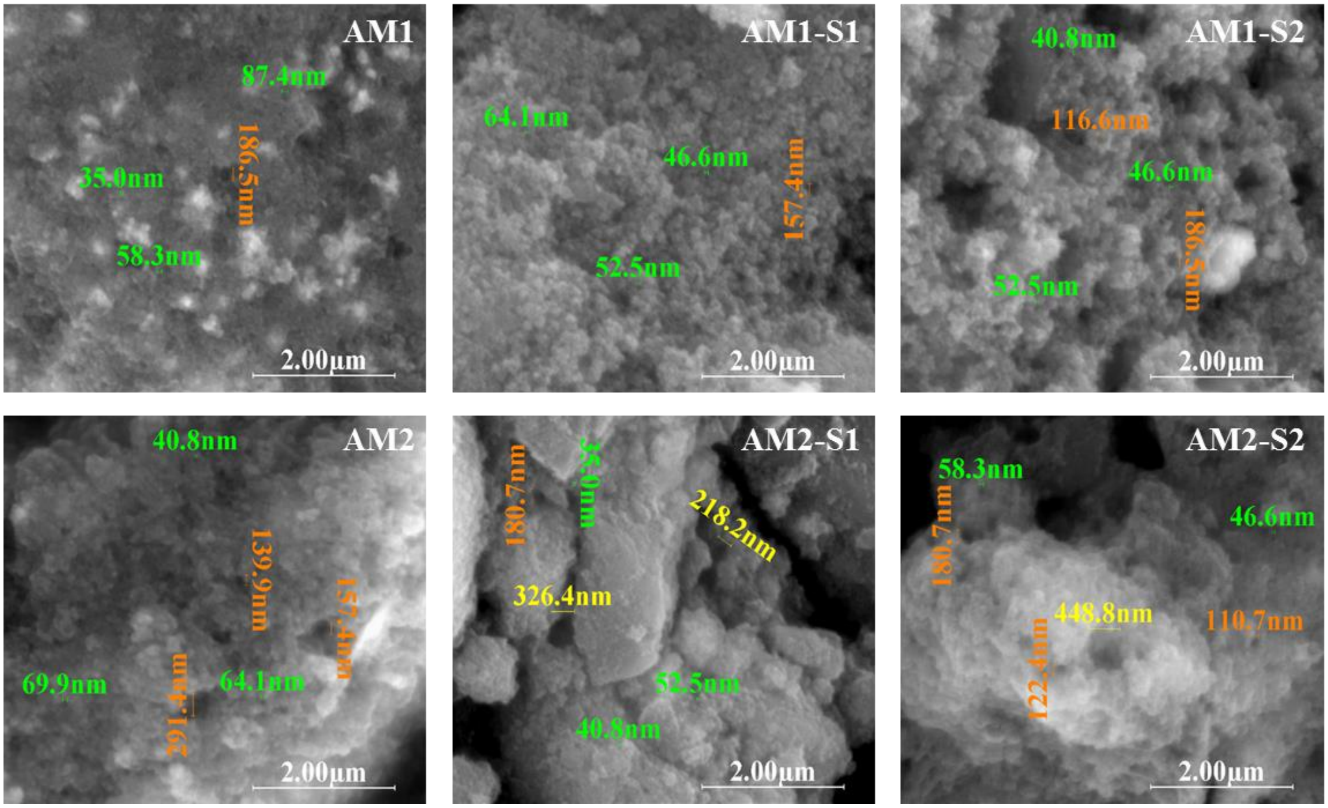

Figure S1. *SEM images with pore sizes* of the Ag-decorated core-shell IOMNPs

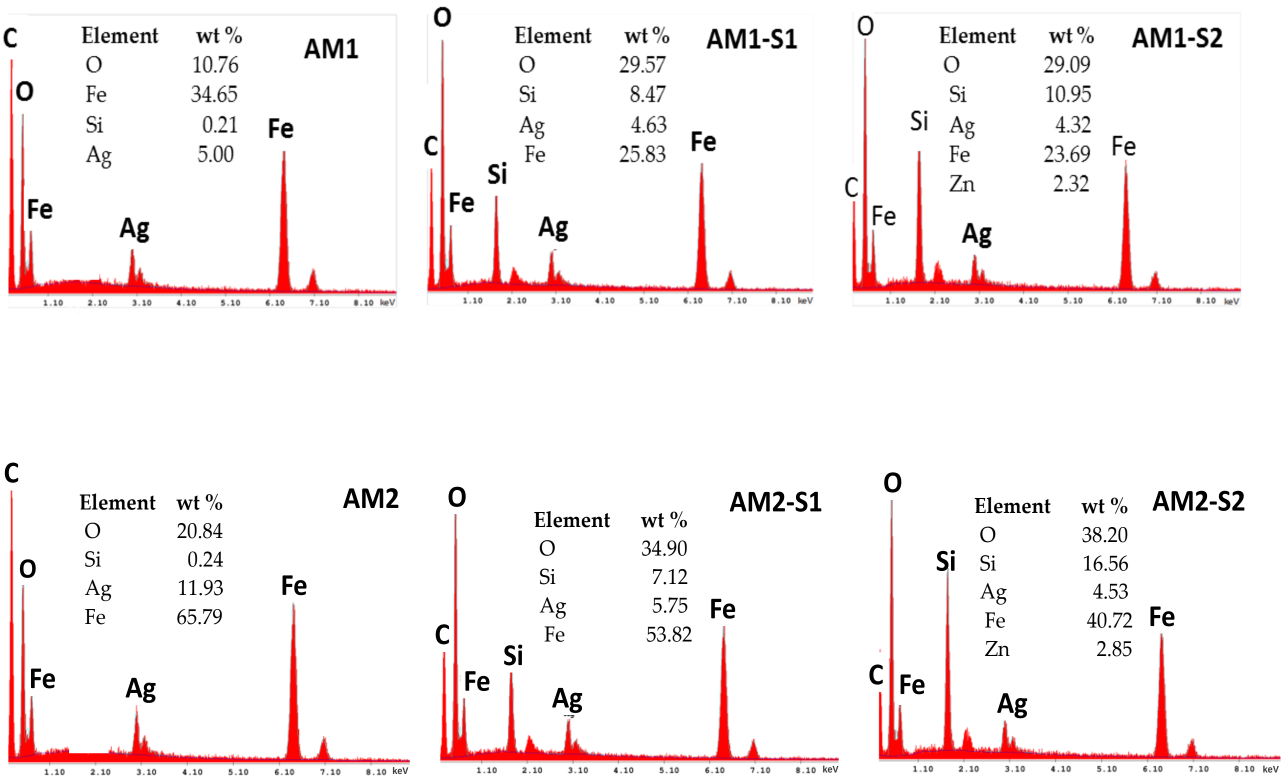

Figure S2. SEM-EDX spectra of the Ag-decorated core-shell IOMNPs

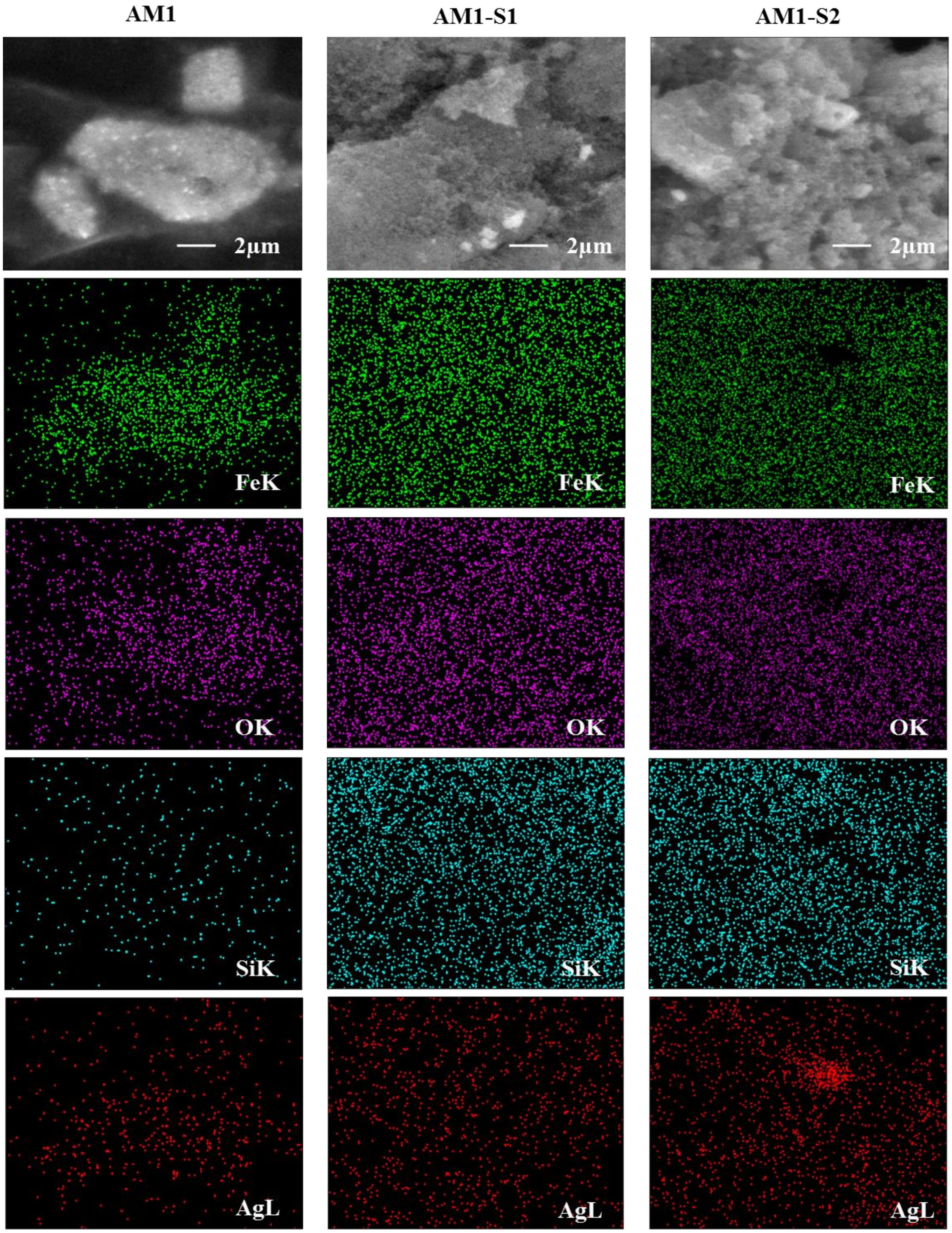
AM1
AM1-S1
AM1-S2
2μm
2μm
2μm
FeK
FeK
FeK
OK
OK
OK
SiK
SiK
SiK
AgL
AgL
AgL

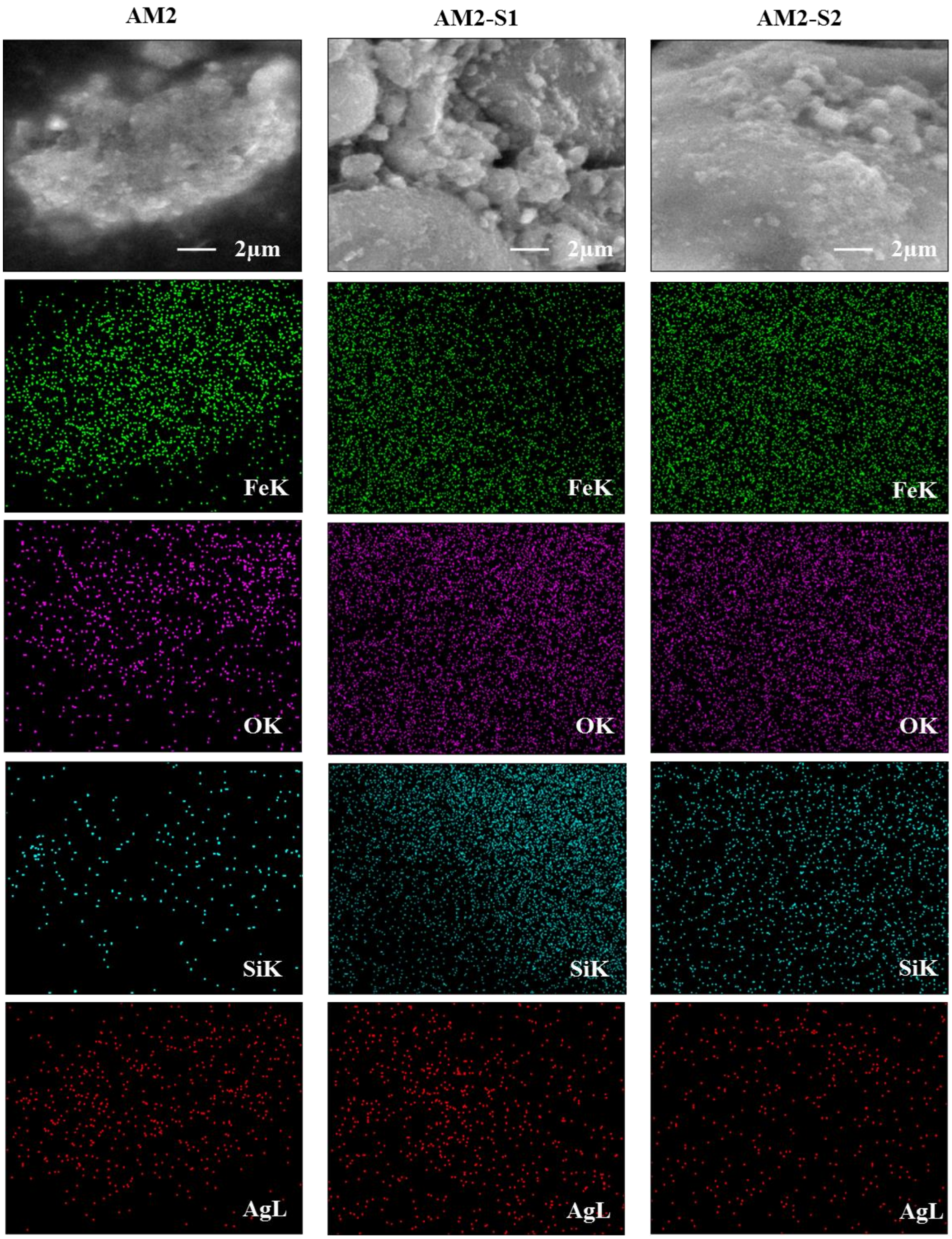

Figure S3. SEM-EDX mapping of the Ag-decorated core-shell IOMNPs

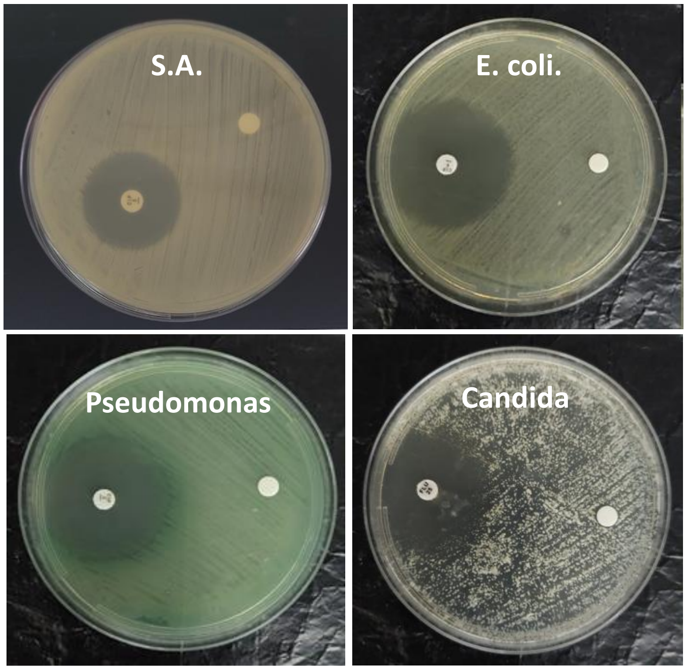

Figure S4. Disk diffusion assay for the control samples carried out on the surface of the inoculated medium: 1 blank 6 mm control disc (negative control), discs with Ciprofloxacin 5 mcg (positive control for *S.A.*, *E. coli* and *Pseudomonas* bacteria) and fluconazole 25 mcg (positive control for *Candida* fungus).

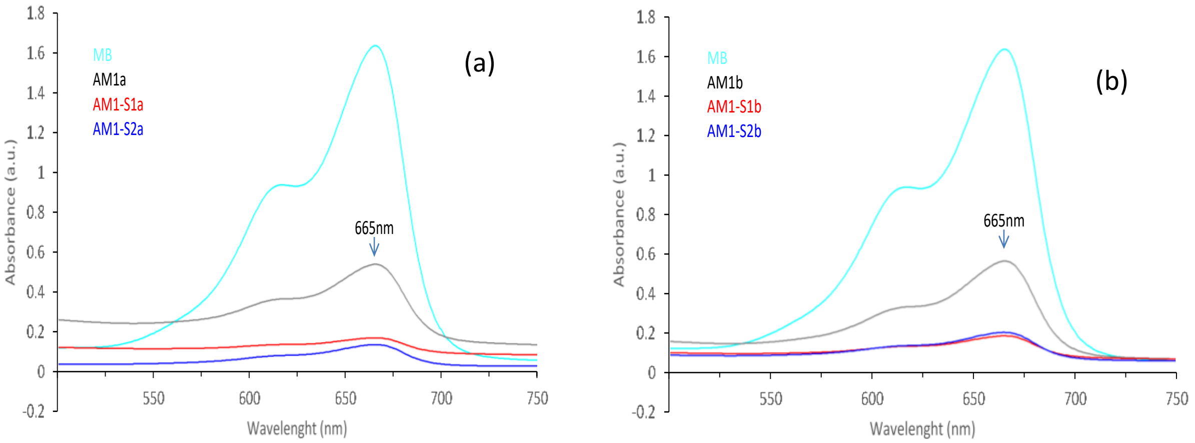

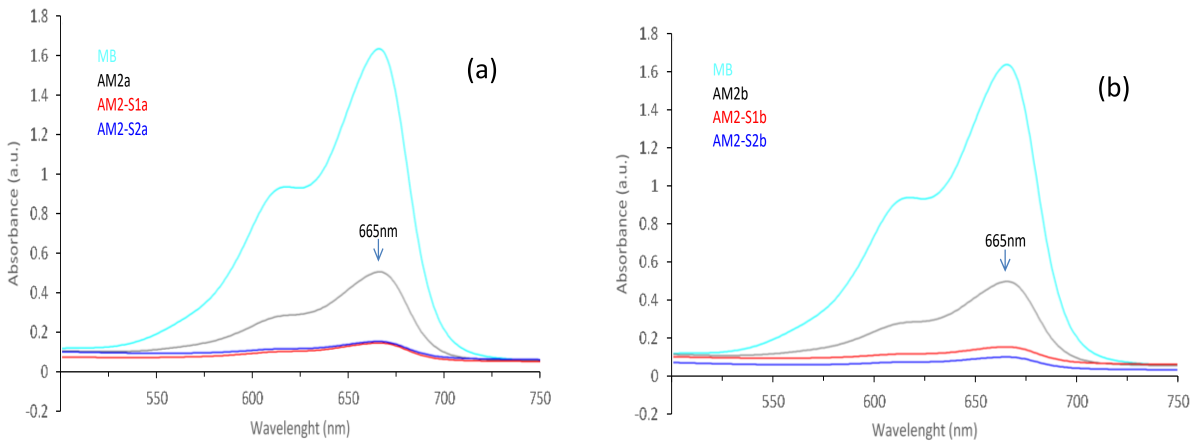

Figure S5. Variation of the intensity of main peak optical absorption of the MB solution after 120 min under UV irradiation (a) or dark (b) without NPs (MB) and in the presence of investigated NPs samples, AM(1/2)-S(1/2)

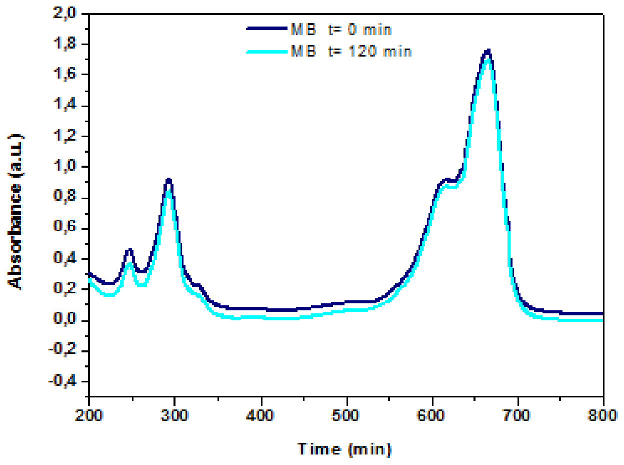

Figure S6. The intensity of the main optical absorption peak of the MB solution, before (t=0 min) and after 120 min under UV irradiation without the presence of investigated samples, used for photocatalytic tests

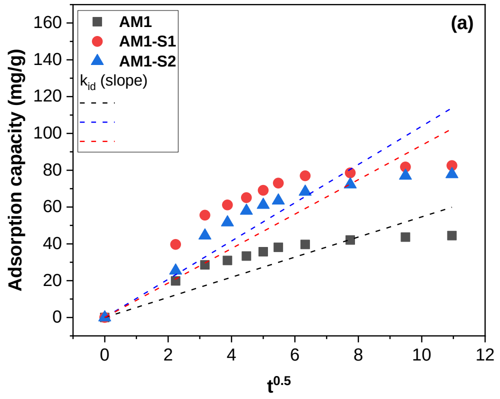

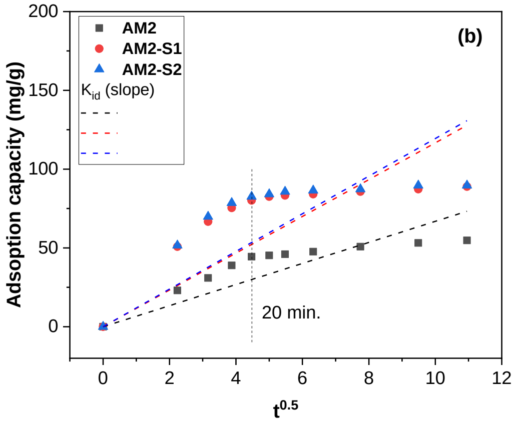

Figure S7. Adsorption capacity versus sqrt (time) and linear fit of MB on the AM1 (a) and Am2 NPs (b)

Table S1 Kinetic parameters (intraparticle diffusion model) of the adsorption process of MB on the surface of the nanocatalysts during 2h

Sample	AM1	AM1-S1	AM1-S2	AM2	AM2-S1	AM2-S2
K_{id}	5.46666±0.44685	10.39155±0.91988	9.35663±0.68067	6.68916±0.54788	11.6674±1.23656	11.94299±1.29384
R2	0.93737	0.92733	0.94974	0.93713	0.89902	0.89492

Table S2. Kinetical parameters of the photocatalysis process of MB degradation under UV irradiation within 0-30 min. range.

**Sample**	**k_0 (min.$^{-1}$)**	**R^2**	**k_1 (min.$^{-1}$)**	**R^2**	**k_2 (mg dm^{-3} min^{-1})**	**R^2**
AM1	0.01938±0.00289	0.88347	0.0252±0.00346	0.89863	0.0261±0.00335	0.91389
AM1-S1	0.03333±0.00421	0.91588	0.06085±0.0047	0.96546	0.10459±0.0021	0.99759
AM1-S2	0.028808±0.00334	0.92162	0.04401±0.00386	0.95586	0.05971±0.0313	0.9838
AM2	0.02058±0.00253	0.91689	0.02746±0.00283	0.94001	0.03005±0.00244	0.96189
AM2-S1	0.0366±0.00532	0.88733	0.07435±0.00757	0.94144	0.15186±0.0725	0.98652
AM2-S2	0.03534±0.00461	0.90754	0.06954±0.00516	0.96807	0.13668±0.00321	0.99669
Equation [1]	$C_t/C_0=1-(k_0/C_0)t$		$ln(C_t/C_0)=k_1t$		$(1/C_t-1/C_0)=k_2t$	

[1] Dagher, S.; Soliman, A.; Ziout, A.; Tit, N.; Hilal-Alnaqbi,A.; Khashan, S.; Alnaimat, F.; Qudeiri, J.A. Photocatalytic removal of methylene blue using titania-and silica-coated magnetic nanoparticles. Mater. Res. Express 2018, 5, 065518. Doi: 10.1088/2053-1591/aacad4
